# Circulating tumor cells share RNA modules with early embryo trophectoderm and with metastatic cancer

**DOI:** 10.1002/cac2.12664

**Published:** 2025-01-23

**Authors:** Stefano Volinia, Anna Terrazzan, Tomasz S. Kaminski, Krystian Jadzewski, Eva Reali, Nicoletta Bianchi, Jeff Palatini

**Affiliations:** ^1^ Department of Translational Medicine Laboratory for Advanced Therapy Technologies University of Ferrara Ferrara Italy; ^2^ Biological and Chemical Research Centre University of Warsaw Warsaw Poland; ^3^ Centre of New Technology University of Warsaw Warsaw Poland; ^4^ Department of Molecular Biology Institute of Biochemistry Faculty of Biology University of Warsaw Warsaw Poland

**Abbreviations**
CTCs: circulating tumor cells
epiA CTC: epithelial A CTC
epiB CTC: epithelial B CTC
LN: metastatic breast cancer lymph nodes
CA: the respective breast cancers related to LN
EVT: extravillous trophoblast
STB: syncytiotrophoblast
CTB: cytotrophoblast
Epi/ICM: epiblast/inner cell mass
Prelin: prelineage
PrE: primitive endoderm
scRNA‐seq: single cell RNA sequencing
TE: trophectoderm
UMAP: Uniform Manifold Approximation and Projection
GRN: gene regulation network
EMT: epithelial to mesenchymal transition
TNBC: triple negative breast cancer
ER^+^
: estrogen receptor‐positive breast cancer
HER2^+^
: human epidermal growth factor receptor 2‐positive breast cancer
CD274: immune checkpoint PD‐L1

1

Metastasis is the primary cause of cancer‐related deaths, accounting for an estimated 66% to 90% of fatalities [1]. It is a multistep process involving the dissemination of circulating tumor cells (CTCs) and their colonization of distant organs [2, 3]. A higher number of detected CTCs in cancer patients is associated with shorter survival [4].

We analyzed 544 single‐cell RNA sequencing (scRNA‐seq) profiles of bona fide CTCs, identified as keratin‐positive and aneuploid, from over 3,000 putative CTC profiles available in public databases, as detailed in Supplementary Table S1. Most of the CTCs originated from patients with breast cancer (*n* = 502, 92.3%), while a smaller number were derived from patients with prostate cancer (*n* = 42). All experimental methods are described in the Supplementary Materials and Methods.

All bona fide CTCs were positive for KRT18 and negative for PTPRC (CD45), as expected. Three main CTC subgroups were identified (Supplementary Figure S1). We labeled the two epithelial (EPCAM^+^) subgroups as epithelial A (epiA) and epithelial B (epiB), while the third subgroup was mesenchymal (VIM^+^/EPCAM^−^). CAV1 and AXL showed the highest specificity for mesenchymal CTCs, whereas LY6E was the most distinctive gene for epiB CTCs (Supplementary Tables S2–S4). Further analysis revealed that mesenchymal and epiB, but not epiA CTCs, were actively engaged in the cell cycle, as inferred using the R package Tricycle (Supplementary Figure S1). The biological implications of these three CTC subgroups are highly relevant. Mesenchymal CTCs expressed significantly lower levels of KRT18 and other keratins, such as KRT19 and KRT7, compared to epithelial CTCs. Conversely, vimentin, another class of intermediate filaments, was highly expressed in mesenchymal CTCs. The shift from keratins to vimentin is a hallmark molecular event in epithelial‐to‐mesenchymal transition (EMT). EMT regulators ZEB1, ZEB2, and SNAI2 were upregulated in mesenchymal CTCs, indicating that EMT was responsible for their origin. These findings highlight the need to prioritize the detection and targeting of epiB and mesenchymal CTCs. PD‐L1 (CD274), an important target for immunotherapy in clinical practice, was expressed in only a small fraction of mesenchymal CTCs and even less in epithelial CTCs (Supplementary Figure S2). In contrast, two other immune checkpoint genes, CD276 (B7‐H3) and PVR (CD155), were highly expressed in CTCs, comparable to their expression in trophoblasts. This suggests an immuno‐evasive phenotype common to most CTCs, driven by the expression of CD276 and PVR.

Is there a functional relationship between CTCs and trophoblast cells, as suggested by the co‐expression of genes such as CD276, SP6, and LY6E (Supplementary Figure S3)? To address this question, we examined potential links between CTCs and the placenta or early embryo by integrating scRNA‐seq profiles of CTCs with those from normal and cancerous breast tissue, early embryos and first‐ and second‐trimester human placenta (Figure 1A, Supplemental Figures S4,S5). The UMAP plot positioned CTCs within a region enclosed by metastatic breast cancer cells, early embryonic cells, and trophoblast cells. We further explored these interrelations using divisive hierarchical spectral clustering (Figure 1B), which confirmed that CTCs, trophoblast cells, and embryonic cells share similar RNA profiles (Supplementary Figure S6 and Supplementary Table S5). To validate these findings, we mapped the CTCs onto the transcriptional landscape of embryo developmental [5] (Figure 1C). A subset of CTCs (*n* = 72 out of 544, *p*‐value < 0.001) aligned with the trophectoderm (TE), the blastocyst cells that give rise to the trophoblast, facilitating embryo attachment and subsequent invasion to form the placenta. Notably, these TE‐like CTCs were predominantly from the epiB subgroup (64 out of 72, Fisher Test *p*‐value < 0.001) and were frequently in the S phase of the cell cycle (Supplementary Figure S7). To further investigate the similarity between epiB CTCs and TE, we performed a transcriptomic correlation analysis across all cell types. In the resulting correlation plot (Figure 1D), epiA CTCs clustered among breast cancer subtypes, while epiB and mesenchymal CTCs clustered with early embryonic stages. In particular, epiB CTCs showed strong similarity to TE and its precursors, pre‐lineage cells. This analysis confirmed the strong relationship between epiB CTCs and the TE lineage previously observed.

**FIGURE 1:**
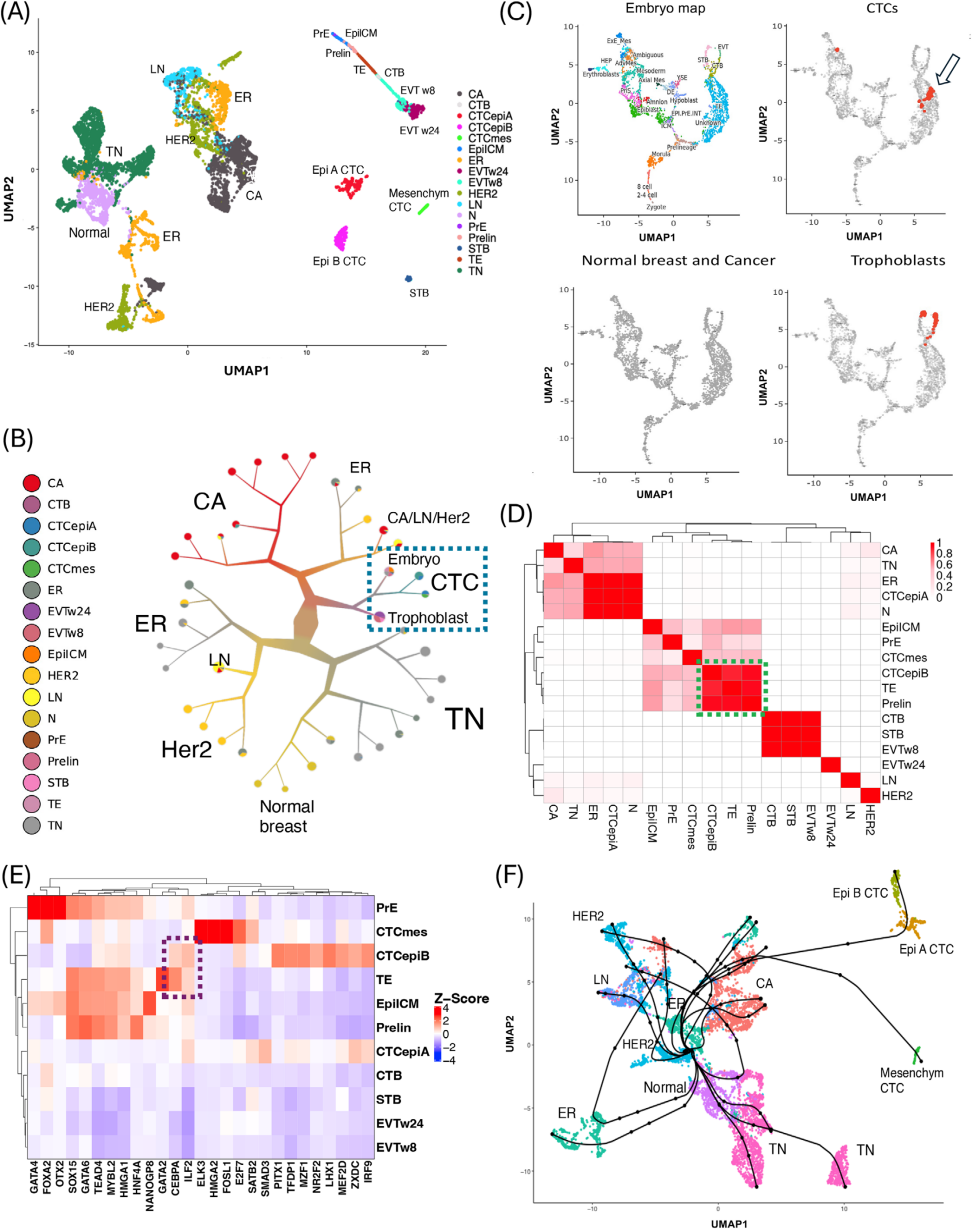
Integration of scRNA‐seq from CTCs, normal and cancer breast samples, and embryonic cell types. (A) UMAP plot of the CTC, cancerous and normal breast tissue, and embryonic cell types upon Scanorama integration. All three CTC subgroups were located in a UMAP space defined by metastatic breast cancers (CA, on the left), EVTs (on the top and right‐hand side), and a small cluster of syncytiotrophoblast (STB, bottom and right‐hand side). The colors in the legend correspond to the different cancer or cell subgroups in the integrated dataset. (B) Divisive hierarchical spectral clustering of CTCs, metastatic and non‐metastatic primary tumors, metastatic lymph nodes, trophoblasts, and embryonic cell types. Nodes were pruned with the “smart prune” set to 0.5 median absolute deviation. Spectral clustering confirmed that CTCs, trophoblasts, and embryo cells possessed similar RNA profiles, as they were clustered together (dashed light blue oval). The color of the clusters and of branches correspond to the relative composition in the different cell types. The cluster size is proportional to its numerosity. (C) CTCs mapped onto the trophectoderm (TE) space in the embryonic development reference map. An interactive online tool based on the human embryogenesis reference was used to map the CTCs onto the human embryo using their scRNA‐seq profiles [5]. The left and top panels show the embryonic reference map with the locations of the major cell types during development. Seventy‐two CTCs (out of 544) mapped to the embryonic trophectoderm (TE), the tissue that originates trophoblasts (positive samples are red dots in the top/right panel, indicated by the arrow). As a positive control, trophoblast cells correctly mapped to their expected area (positive samples are red dots in the bottom/right panel). Conversely, in negative control tests (bottom/left panel), no cells from non‐metastatic cancer (400 samples each for TNBC, ER^+^, and HER2^+^ breast cancer subtypes) or normal breast epithelial cells (*n* = 703) were classified as TE or other embryonic cell types. Thus, the CTCs had a highly significant excess of cells similar to TEs (chi‐square test, *p*‐value < 0.001). The TE‐like CTCs predominantly belonged to the CTC epithelial B subgroup (64 out of 72, Fisher Test *p*‐value < 0.001) and were frequently in the S phase of the cell cycle (Supplementary Figure S7). (D) The correlation matrix shows that the transcriptome of epithelial B CTCs has the strongest similarities with those of trophectoderm (TE) and prelineage embryonic cells (Prelin). We performed a correlation analysis of the aggregate expression for all cell types, including CTCs, normal and cancerous breast samples, early embryos, and trophoblasts, in the integrated dataset. In the correlation plot, epithelial A CTCs clustered among the cancer subtypes, while epithelial B and mesenchymal CTCs clustered among early embryonic stages. In particular, epithelial B CTCs showed the highest similarity with TE and its precursor prelineage cells (green oval). (E) Active gene regulatory networks (GRNs) in CTCs and embryonic cell types. We used single‐cell regulatory network inference and clustering (SCENIC) to reconstruct gene regulatory networks [10]. EpiB and mesenchymal CTCs displayed divergent transcription programs and various specific GRNs. Only epiB CTCs shared regulation by the master gene CEBPA with TE (dashed dark blue oval). When considering activated GRNs, epiB CTCs clustered with TE and early embryonic cell types. (F) We investigated the paths and lineages leading from normal breast epithelial cells through the various breast cancer subtypes to metastatic lymph nodes and CTCs. The inferred pseudo‐time and reconstructed lineages were obtained using Slingshot [6] and the minimum spanning tree. The normal breast clusters were correctly selected as the starting points by unsupervised analysis, ending with the epithelial A/B CTCs via cancer cell clusters from ER^+^ and metastatic tumors. The mesenchymal CTCs followed a late diverging trajectory. The lineages for the breast cancer progression, except for the TNBC subtype, included some common evolutionary segments preceding the emergency of CTCs. Abbreviations: CTC, circulating tumor cell; CTCepiA, epithelial A CTC; CTCepiB, epithelial B CTC; CTCmes, mesenchymal CTC; N, normal breast; LN, metastatic breast cancer lymph nodes; CA, the respective breast cancers related to LN; EVTw8, extravillous trophoblast at 8 weeks; EVTw24, extravillous trophoblast at 24 weeks; STB, syncytiotrophoblast; CTB, cytotrophoblast; Epi/ICM, epiblast/inner cell mass; Prelin, prelineage; PrE, primitive endoderm; TE, trophectoderm; TN, triple negative breast cancer; ER, estrogen receptor‐positive breast cancer; HER2, human epidermal growth factor receptor 2‐positive breast cancer; scRNA‐seq, single cell RNA sequencing; UMAP, Uniform Manifold Approximation and Projection; GRN, gene regulation network.

We hypothesized that the similarity between epiB CTCs and early embryonic stages arises from functional convergence. Specifically, we proposed that key traits essential for the functionality of CTCs (invasiveness and immune‐evasion) are encoded in the human genome as part of the trophectodermal program, which leads to the extravillous trophoblast and ultimately to the placenta. To test this hypothesis, we investigated transcription factors that were upregulated and had active gene regulatory networks (GRNs) in both epiB CTCs and TE or its precursors, pre‐lineages (Figure 1E). While epiB CTCs exhibited several active GRNs, only CEBPA and ILF2 were shared with TE. Furthermore, while ILF2 was ubiquitously expressed across the dataset, the upregulation of CEBPA mRNA was predominantly restricted to epiB CTCs and TE (Supplementary Figure S8). Additionally, we identified several GRNs that appeared to be specific to either epiB or mesenchymal CTCs (Figure 1E).

Is it possible to define a cellular path for cancer establishment and progression in breast cancer, given the diversity of the scRNA‐seq profiles in the dataset we assembled? To reconstruct the lineages leading from normal breast tissue, through various breast cancer subtypes, to metastatic lymph nodes and eventually to CTCs, we inferred pseudotime using Slingshot [6]. Although the analysis was unsupervised, the normal breast clusters were accurately identified as the starting points, culminating in the epithelial A/B CTCs via intermediate cell clusters from ER^+^ and metastatic cancers (Figure 1F). The progression lineages for ER^+^ and HER2^+^ breast cancer shared common evolutionary segments, ultimately leading to the emergence of CTCs (Supplementary Table S6).

We finally identified RNA modules that could be relevant to metastatic evolution. The genes upregulated in CTCs, metastatic lymph nodes, and their respective primary tumors are shown in Supplementary Figure S9 and listed in Supplementary Table S7. Two genes associated with metastasis (ALDOA and PSMA6) were also upregulated in TE. The expression levels of RNA modules implicated in progression are displayed, superimposed on the UMAP plot, in Supplementary Figures S10–S12.

In this study, we aimed to characterize CTCs within the context of the cancer environment using scRNA‐seq. We identified a mesenchymal CTC (VIM^+^/AXL1^+^) subpopulation, distinct from the larger epithelial CTCs (EPCAM^+^) population. Importantly, we further divided the epithelial CTCs into two divergent subgroups: epiA, characterized by high CD24/CDH1 expression, and epiB, marked by elevated levels of the stem cell master regulators SOX2/CEBPA. Notably, epiB and mesenchymal CTCs, but not epiA CTCs, exhibited mitotic activity. Of clinical significance, CD276 and PVR, but not PD‐L1, were the primary immune checkpoint genes expressed in CTCs. CD276, like PD‐L1, is an immune checkpoint that suppresses tumor antigen‐specific immune responses and is a target of anticancer agents such as enoblituzumab [7], and CAR T cells [8]. We propose that CD276 and PVR could serve as targets for novel immunotherapeutic strategies to eliminate CTCs.

In conclusion, we identified a novel CTC subtype, epiB, along the lineages of breast cancer progression, characterized by high levels of the stem cell master regulator CEBPA and significant mitotic activity. For the first time, we also demonstrated a link between this CTC subgroup, epiB, and the embryonic trophectoderm. EpiB CTCs may utilize elements of the TE genetic program to invade the vasculature, achieve metastasis, and implement fetal‐like immune tolerance. The RNA modules involved in cancer progression that we identified, particularly those of mesenchymal and epithelial B CTCs, could have clinical applications in detecting minimal residual disease [9] and in identifying novel molecular targets in metastasis.

## AUTHOR CONTRIBUTIONS

Stefano Volinia conceived and designed the study, collected the data, and performed the analysis. Stefano Volinia, Krystian Jazdzewski, Anna Terrazzan, Jeff Palatini, Tomasz S Kaminski, Eva Reali, and Nicoletta Bianchi discussed and revised the methods and results. Stefano Volinia, Krystian Jazdzewski, Anna Terrazzan, Jeff Palatini, Tomasz S. Kaminski, Eva Reali, and Nicoletta Bianchi drafted the manuscript. All authors read, revised, and approved the final manuscript.

## CONFLICT OF INTEREST STATEMENT

The authors declared no potential conflicts of interest with respect to the research, authorship, and/or publication of this article.

## FUNDING INFORMATION

Italy's MUR PNRR National Center for HPC, big data and quantum computing (CN00000013 CN1) and Poland's National Science Centre project OPUS 24 (2022/47/B/NZ7/03418) to Stefano Volinia. Stefano Volinia was also recipient of a Polish NAWA Ulam Scholarship (BPN/ULM/2021/1/00232) and of an University of Ferrara FAR 2024 grant. Krystian Jazdzewski was supported by Foundation for Polish Science (POIR.04.04.00‐00‐1DD9/16‐00).

## ETHICS APPROVAL AND CONSENT TO PARTICIPATE

We confirm that all methods were carried out in accordance with relevant guidelines and regulations. Data were obtained from public databases.

## Supporting information



Supporting Information

Supporting Information

## Data Availability

All data needed to evaluate the conclusions in the paper are present in the paper and/or in the Supplementary Materials. Processed single‐cell gene expression data are available to download at Zenodo (https://doi.org/10.5281/zenodo.13762250).
