# Correction to “Circulating tumor cells share RNA modules with early embryo trophectoderm and with metastatic cancer”

**DOI:** 10.1002/cac2.70062

**Published:** 2025-09-26

Volinia S, Terrazzan A, Kaminski TS, Jazdzewski K, Reali E, Bianchi N, Palatini J. Circulating tumor cells share RNA modules with early embryo trophectoderm and with metastatic cancer. *Cancer Commun (Lond)*. 2025;45(5):500‐504. doi:10.1002/cac2.12664

We spelled an author name incorrectly. We kindly request a change to the spelling:

Author: Jadzewski (wrong) to Jazdzewski (correct), adding the “z”, in the author list.

We apologize for this error.

