# Supplementary material for: Circulating tumor cells share RNA modules with early embryo trophectoderm and with metastatic cancer
Source: Cancer Commun (Lond). 2025 Jan 23;45(5):500–4. doi: 10.1002/cac2.12664 (PMC12067392; doi:10.1002/cac2.12664)
Supplement: Supplementary file 2 — Supporting Information [file CAC2-45-500-s002.docx]

**Supplementary Materials**

**Circulating tumor cells share RNA modules with early embryo trophectoderm and with metastatic cancer**

Stefano Volinia^1,2,3,*^, Anna Terrazzan^1^, Tomasz S. Kaminski^4^, Krystian Jadzewski^2^, Eva Reali^1^, Nicoletta Bianchi^1,$^, Jeff Palatini^3,$^

^1^Department of Translational Medicine, Laboratory for Advanced Therapy Technologies, University of Ferrara, Ferrara 44121, Italy.

^2^Biological and Chemical Research Centre, University of Warsaw, Warsaw 02-089, Poland.

^3^Centre of New Technology, University of Warsaw, Warsaw 02-097, Poland.

^4^Department of Molecular Biology, Institute of Biochemistry, Faculty of Biology, University of Warsaw, Warsaw 02-096, Poland.

^$^Nicoletta Bianchi and Jeff Palatini contributed equally to this work.

^*^Corresponding author:

Stefano Volinia, Department of Translational Medicine, Laboratory for Advanced Therapy Technologies, University of Ferrara, Via Fossato di Mortara 70, Ferrara 44121, Italy. Email: [s.volinia@unife.it](mailto:s.volinia@unife.it).

**Supplementary Materials and Methods**

Single-cell RNA-seq (scRNA-seq) profiles of bona-fide keratin-positive and aneuploid CTCs were identified among putative CTCs deposited in the GEO and SRA databases and are listed in **Supplementary Table S1**. Informed consent is not required because the study accessed anonymized data from public databases. Single-cell profiles for ER^+^, HER2^+^, and TN breast cancer were obtained from GSE161529 [1,2]. Additionally, we obtained single-cell profiles of primary and paired metastatic lymph node tumors in breast cancer patients [3] (GSE167036). Normal breast profiles were obtained from the HBCA project [4]. The human breast cell atlas, assembled from 55 donors who underwent reduction mammoplasties or risk reduction mastectomies, was used for the single cell normal breast data [5]. Single-cell profiles of sorted placental cells from first- and second-trimester human placentas were obtained from GEO (GSE89497) [6]. spanning cytotrophoblast cells (CTBs), extra-villous trophoblast cells (EVTs), Hofbauer cells, and mesenchymal stromal cells. When raw counts were not available, FASTQ files were aligned using STAR [7].

Scevan [8] and CopyKAT [9] R packages were used for the identification of CNV and aneuploidy in cancer cells. The complex and highly heterogeneous single-cell profiles from tumors and lymph nodes underwent classification and purification stages. Specifically, blood-derived cells (i.e., those in clusters showing high expression of CD45/PTPRC, CD52, CXCR4, or CD74) were removed from cancer tissues, while endothelial cells were identified based on their positivity for P-selectin (SELP) or von Willebrand factor (VWF). To minimize influences of mitotic activity on cell clustering, we removed cell cycle genes listed in **Supplementary Table S8**. These genes are well-established cell cycle markers [10] (*n* = 97), which were loaded into Seurat and categorized into markers of the G2/M and S phases. Single-cell profiles, including all genes measured in each independent dataset, were processed to implement quality control (nFeatures > 2,500 and nCount > 10,000). The various datasets were integrated using Scanorama [11]. A global annotation was obtained using the mammary gland reference from Tabula Sapiens and popv [12]. Cells of hematopoietic lineage were removed from primary tumors, lymph nodes, and controls. Tumor stromal cells, such as endothelial cells (TEC), PVLs, CAFs, and other non-Athelial cells, were also removed. The filtered dataset primarily contained epithelial cells from normal and cancerous breast tissues, as well as from CTCs. Notably, most of the mesenchymal-like CTCs (of breast origin) were retained after the removal of non-epithelial cells, whereas those from pancreas were not. Prostate CTCs were also retained as epithelial cells.

The R package Tricycle [13] was employed to infer and visualize cell cycle positions based on the default gene list (RevelioGeneList). This five-stage cell cycle gene marker list originates from the Revelio package and includes five vectors corresponding to highly expressed genes at each cell cycle stage, as originally reported by Whitfield et al. [14]. The Human Protein Atlas [15] was referenced for cell type-specific expression and subcellular protein localization (https://[www.proteinatlas.org](http://www.proteinatlas.org/), accessed on September 3, 2024). Gene markers were selected using the Wilcoxon test coupled with ROC and AUC analyses [16,17], after transforming raw counts using shifted log [18]. For cluster resolution, we tested both the Louvain (default in Seurat FindCluster function) and Leiden algorithms to assess any divergence in downstream analysis. To choose the optimal K, we focused on achieving the best separation of embryonic cell types and selected K = 5. The Louvain and Leiden algorithms were very similar, as shown in **Supplementary Figure S4** and **Supplementary Tables S9-S11**. Reassuringly, all CTC clusters (*n* = 8) were identical when comparing the results of the Leiden algorithm with those of the Louvain algorithm. Nevertheless, the Leiden algorithm performed better for the separation of early embryonic stages by splitting the Epi/ICM and Prelin cell types, which were grouped as a single mixed cluster (#33) using Louvain, into two distinct clusters (#46 and #47) using Leiden. Based on these findings, we used the Leiden algorithm as the primary clustering method. As an alternative clustering strategy, we also used divisive hierarchical spectral clustering [19]. Spectral clustering was performed on shifted log transformation, and the nodes were pruned with smart prune set to 0.5 MAD. We used ShinyEmbryo [20] as a tool to map CTCs onto the embryonic developmental RNA map.

Regulons were inferred using pySCENIC in Python [21]. First, the gene regulatory network for each sample was constructed using the “grn” function on loom files generated by Seurat and the human (hg38) transcription factor list. Then, the regulons were identified using the “ctx” function, and the “aucell” function was used to compute the area under each curve, representing the activity of each regulon. To obtain cell type-specific rankings of regulons, regulon specificity scores were computed for each regulon and cell type. Regulon activities were visualized for each cell type using z-score and ComplexHeatmap package in R.

To reconstruct the lineages in pseudo-time, starting from normal breast epithelial cells and progressing through various breast cancer subtypes to metastatic lymph nodes and CTCs, we used Slingshot [22] and the minimum spanning tree with unsupervised analysis. UCell was employed to score small RNA modules using the Mann-Whitney U statistic, depending only on the relative gene expression in each cell [23].

R, Bioconductor [24], and RStudio (Posit Software, PBC, Boston, MA, USA) were used for the scRNA-seq analysis described above, alongside Python and Scanpy [25].

**Supplementary references**

1. Pal B, Chen Y, Vaillant F, Capaldo BD, Joyce R, Song X, et al. A single-cell RNA expression atlas of normal, preneoplastic and tumorigenic states in the human breast. EMBO J. 2021;40:e107333.
2. Chen Y, Pal B, Lindeman GJ, Visvader JE, Smyth GK. R code and downstream analysis objects for the scRNA-seq atlas of normal and tumorigenic human breast tissue. Sci Data. 2022;9:96.
3. Liu T, Liu C, Yan M, Zhang L, Zhang J, Xiao M, et al. Single cell profiling of primary and paired metastatic lymph node tumors in breast cancer patients. Nat Commun. 2022;13:6823.
4. Kumar T, Nee K, Wei R, He S, Nguyen QH, Bai S, et al. A spatially resolved single-cell genomic atlas of the adult human breast. Nature. 2023;620:181–91.
5. Reed AD, Pensa S, Steif A, Stenning J, Kunz DJ, Porter LJ, et al. A single-cell atlas enables mapping of homeostatic cellular shifts in the adult human breast. Nat Genet. 2024;1–11.
6. Liu Y, Fan X, Wang R, Lu X, Dang Y-L, Wang H, et al. Single-cell RNA-seq reveals the diversity of trophoblast subtypes and patterns of differentiation in the human placenta. Cell Res. 2018;28:819–32.
7. Dobin A, Davis CA, Schlesinger F, Drenkow J, Zaleski C, Jha S, et al. STAR: ultrafast universal RNA-seq aligner. Bioinformatics. 2013;29:15–21.
8. De Falco A, Caruso F, Su X-D, Iavarone A, Ceccarelli M. A variational algorithm to detect the clonal copy number substructure of tumors from scRNA-seq data. Nat Commun. 2023;14:1074.
9. Gao R, Bai S, Henderson YC, Lin Y, Schalck A, Yan Y, et al. Delineating copy number and clonal substructure in human tumors from single-cell transcriptomes. Nat Biotechnol. 2021;39:599–608.
10. Tirosh I, Izar B, Prakadan SM, Wadsworth MH, Treacy D, Trombetta JJ, et al. Dissecting the multicellular ecosystem of metastatic melanoma by single-cell RNA-seq. Science. 2016;352:189–96.
11. Luecken MD, Büttner M, Chaichoompu K, Danese A, Interlandi M, Mueller MF, et al. Benchmarking atlas-level data integration in single-cell genomics. Nat Methods. 2022;19:41–50.
12. THE TABULA SAPIENS CONSORTIUM. The Tabula Sapiens: A multiple-organ, single- cell transcriptomic atlas of humans. Science. 2022;376:eabl4896.
13. Zheng SC, Stein-O’Brien G, Augustin JJ, Slosberg J, Carosso GA, Winer B, et al. Universal prediction of cell-cycle position using transfer learning. Genome Biology. 2022;23:41.
14. Whitfield ML, Sherlock G, Saldanha AJ, Murray JI, Ball CA, Alexander KE, et al. Identification of genes periodically expressed in the human cell cycle and their expression in tumors. Mol Biol Cell. 2002;13:1977–2000.
15. Karlsson M, Zhang C, Méar L, Zhong W, Digre A, Katona B, et al. A single–cell type transcriptomics map of human tissues. Science Advances. 2021;7:eabh2169.
16. Li Y, Ge X, Peng F, Li W, Li JJ. Exaggerated false positives by popular differential expression methods when analyzing human population samples. Genome Biol. 2022;23:79.
17. Korsunsky I, Nathan A, Millard N, Raychaudhuri S. Presto scales Wilcoxon and auROC analyses to millions of observations [Internet]. bioRxiv; 2019 [cited 2023 Dec 2]. p. 653253. Available from: https://[www.biorxiv.org/content/10.1101/653253v1](http://www.biorxiv.org/content/10.1101/653253v1)
18. Ahlmann-Eltze C, Huber W. Comparison of transformations for single-cell RNA-seq data. Nat Methods. 2023;20:665–72.
19. Schwartz GW, Zhou Y, Petrovic J, Fasolino M, Xu L, Shaffer SM, et al. TooManyCells identifies and visualizes relationships of single-cell clades. Nat Methods. 2020;17:405–13.
20. Zhao C, Plaza Reyes A, Schell JP, Weltner J, Ortega NM, Zheng Y, et al. A comprehensive human embryo reference tool using single-cell RNA-sequencing data. Nat Methods. 2024;1–14.
21. Kumar N, Mishra B, Athar M, Mukhtar S. Inference of Gene Regulatory Network from Single-Cell Transcriptomic Data Using pySCENIC. Methods Mol Biol. 2021;2328:171–82.
22. Street K, Risso D, Fletcher RB, Das D, Ngai J, Yosef N, et al. Slingshot: cell lineage and pseudotime inference for single-cell transcriptomics. BMC Genomics. 2018;19:477.
23. Andreatta M, Carmona SJ. UCell: Robust and scalable single-cell gene signature scoring. Computational and Structural Biotechnology Journal. 2021;19:3796–8.
24. Huber W, Carey VJ, Gentleman R, Anders S, Carlson M, Carvalho BS, et al. Orchestrating high-throughput genomic analysis with Bioconductor. Nat Methods. 2015;12:115–21.
25. Wolf FA, Angerer P, Theis FJ. SCANPY: large-scale single-cell gene expression data analysis. Genome Biology. 2018;19:15.


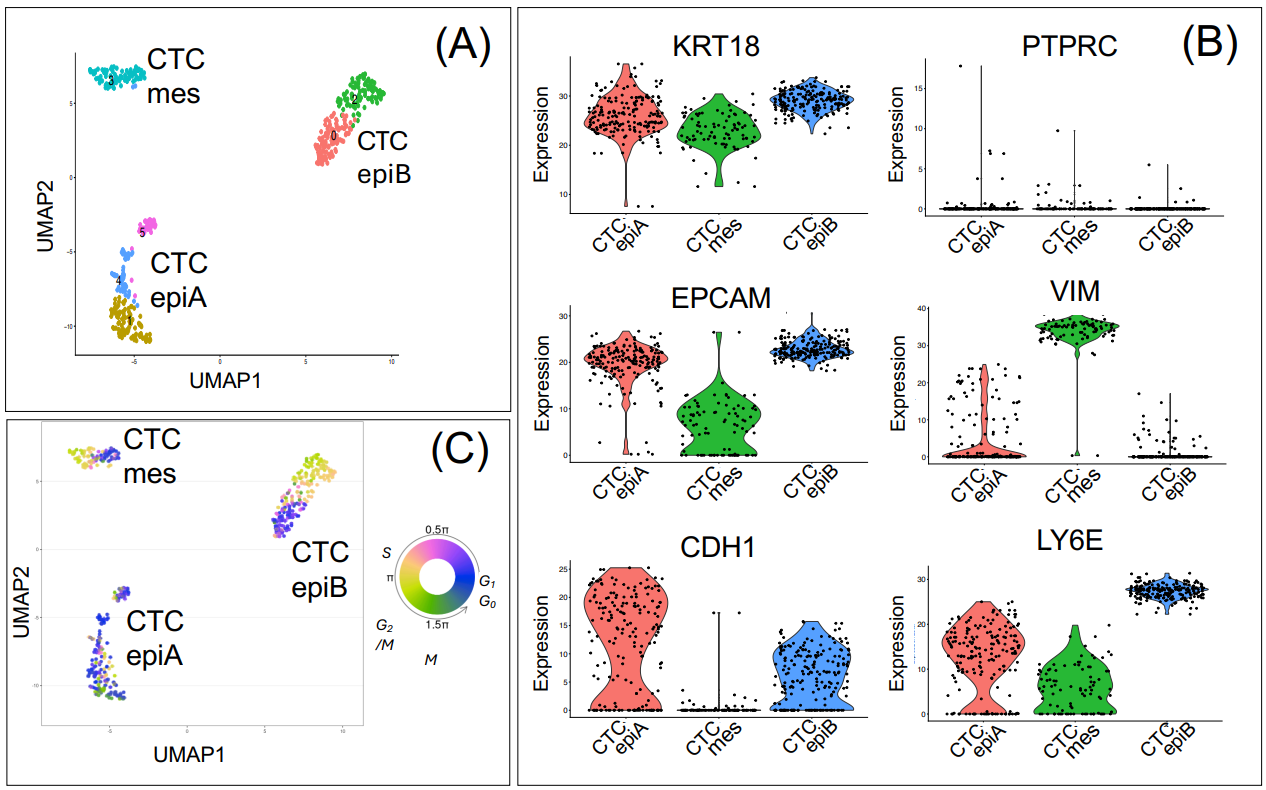


**Supplementary Figure S1.** Single-cell RNA-seq profiling of CTCs identifies three subgroups with diverging molecular phenotypes and mitotic activities.

(A) The bona fide CTCs (*n* = 544) were subdivided into three subgroups: Epithelial A, Epithelial B and mesenchymal CTCs. The UMAP representation shows the separation of the three CTC subgroups into six clusters (#0-5) based on scRNA-seq profiles.

(B) Violin plot displaying the expression levels of canonical CTC markers in the three CTC subgroups. All CTCs expressed KRT18, as expected for cells derived from solid cancers. VIM was upregulated in the mesenchymal subtype, while high levels of LY6E distinguished epithelial A from epithelial B CTCs. No PTPRC (CD45) expression was detected in most CTCs, consistent with its role as a leukocyte marker.

(C) The cell cycle was activated in mesenchymal cells (cluster #3) and a large fraction of epithelial B CTCs (mostly in cluster #2), here superimposed on the UMAP plot. The tricycle R package was used to infer cell cycle position of each cell based on its RNA profile.

Abbreviations: CTC: circulating tumor cell; CTCepiA: Epithelial A CTCs; CTCepiB: Epithelial B CTCs; CTCmes: mesenchymal CTCs.


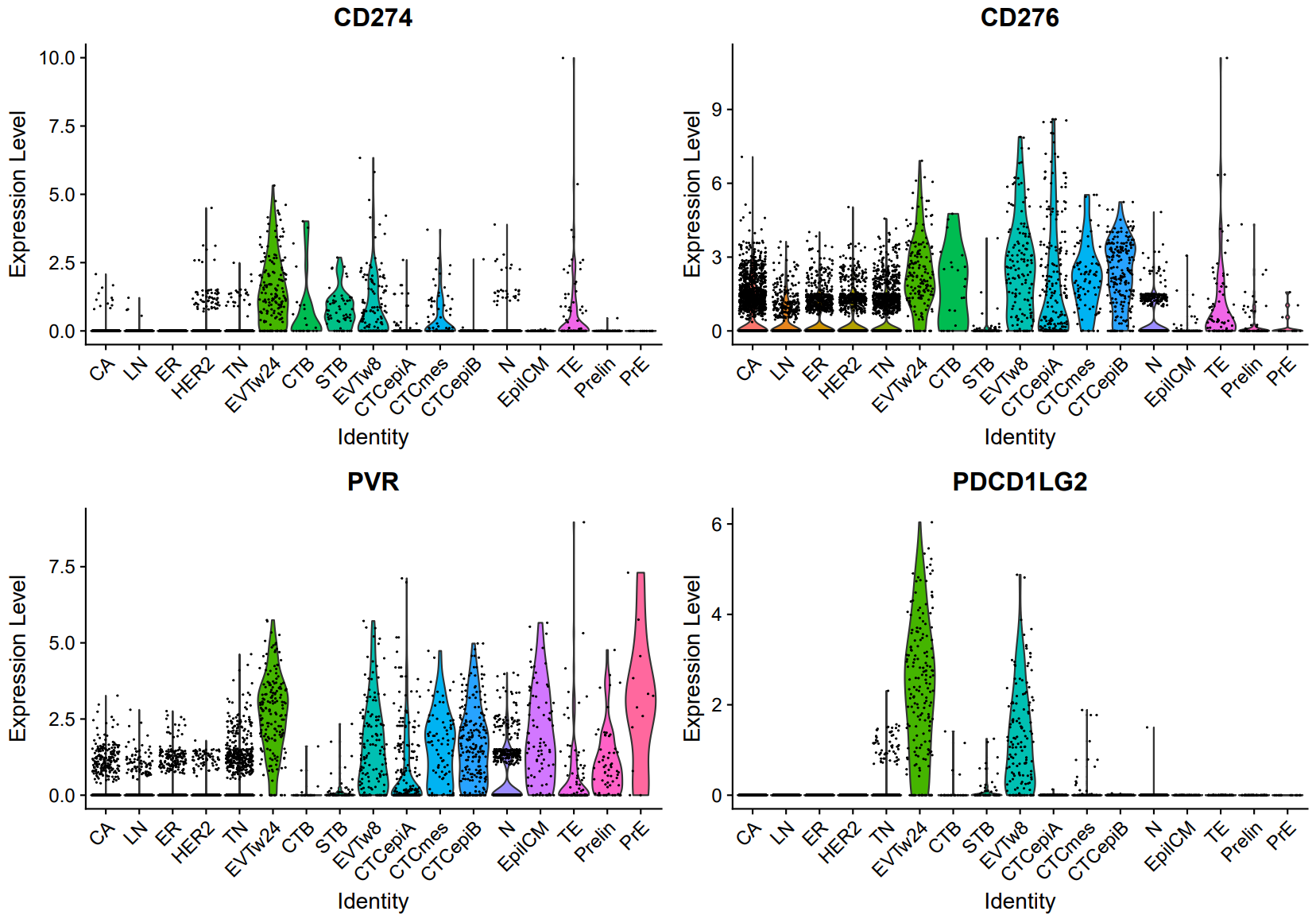


**Supplementary Figure S2.** **Expression of immune check point genes in CTC subgroups, early embryos, and trophoblasts.**

CD274 (PD-L1), an important target for immunotherapy in clinical practice, was expressed in only a small fraction of mesenchymal CTCs and even less in epithelial CTCs. PDCD1LG2 (PD-L2) was not expressed in CTCs. Two other immune checkpoint genes, CD276 (B7-H3) and PVR (CD155), were expressed in CTCs.

Abbreviations: CTC: circulating tumor cell; CTCepiA: epithelial A CTC; CTCepiB: epithelial B CTC; CTCmes: mesenchymal CTC; N: normal breast; LN: metastatic breast cancer lymph nodes; CA: the respective breast cancers related to LN; EVTw8: extravillous trophoblast at 8 weeks; EVTw24: extravillous trophoblast at 24 weeks; STB: syncytiotrophoblast; CTB: cytotrophoblast; Epi/ICM: epiblast/inner cell mass; Prelin: prelineage; PrE: primitive endoderm; TE: trophectoderm; TN, triple negative breast cancer; ER, estrogen receptor-positive breast cancer; HER2, human epidermal growth factor receptor 2-positive breast cancer; UMAP: Uniform Manifold Approximation and Projection.


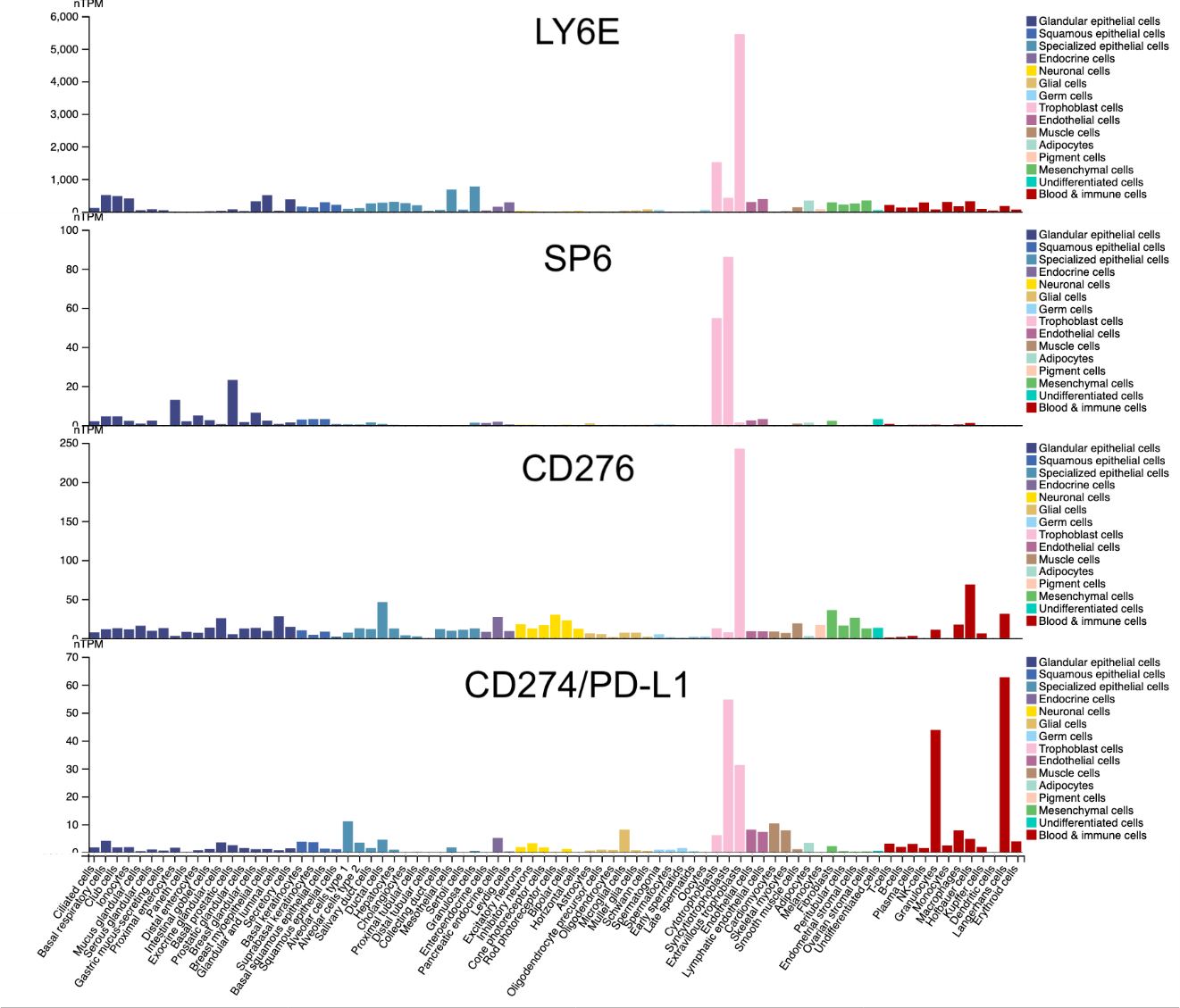


**Supplementary Figure S3.** **LY6E, SP6, and CD276, three markers for CTCs, show high expression in trophoblasts across human tissues.**

PD-L1/CD274 is shown for comparison, with lower expression in trophoblasts but higher expression in granulocytes and Langerhans cells. Hofbauer cells, which express the highest levels of CD276 among hemopoietic cells, are physically associated with the placenta. Results were obtained from the Protein Atlas (https://[www.proteinatlas.org)](http://www.proteinatlas.org/) on 3rd September 2024)


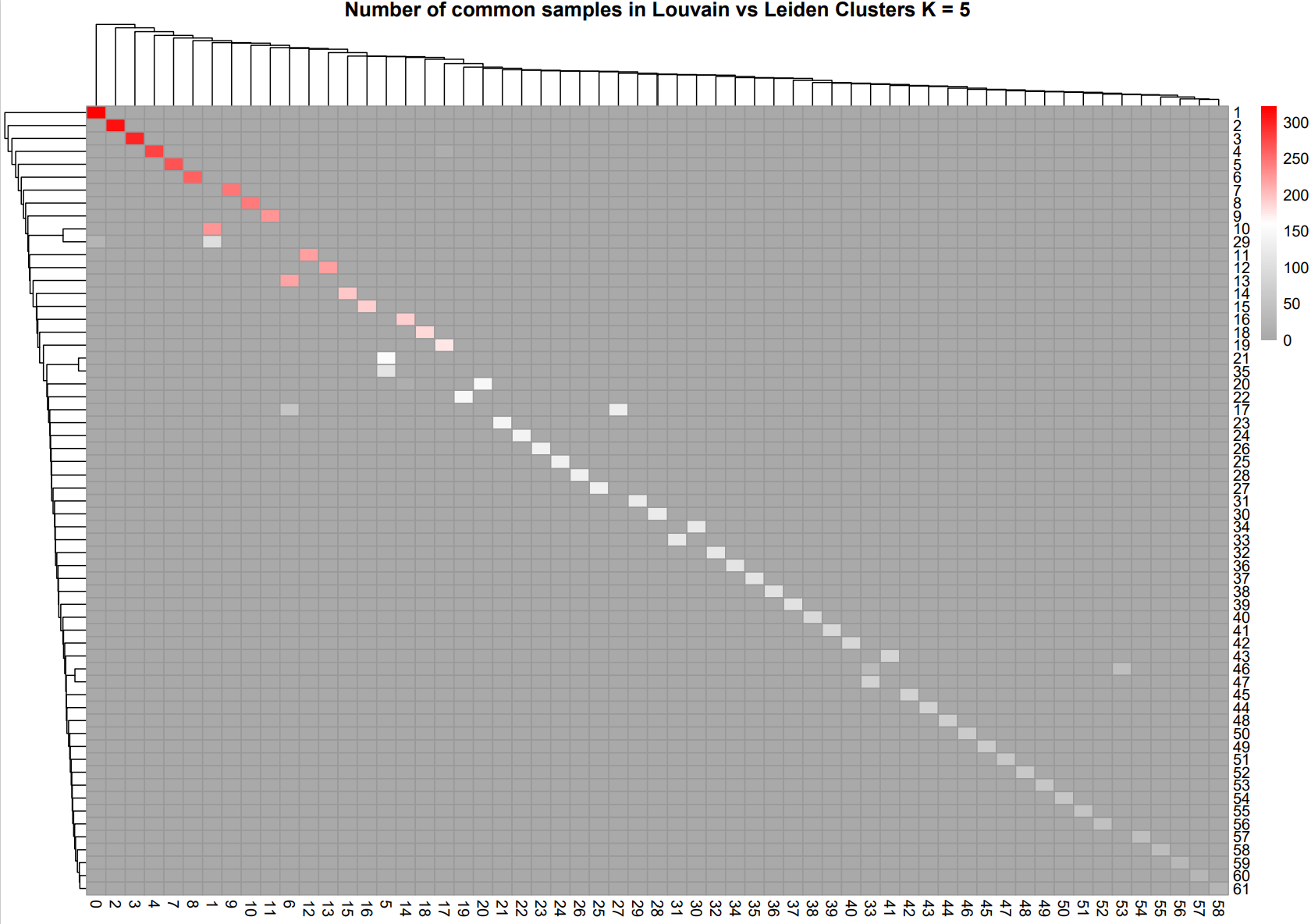


**Supplementary Figure S4. Comparison between Leiden and Louvain clustering algorithms in the integrated dataset.**

The dataset was divided into clusters (K = 5) after Scanorama integration, using the FindClusters function in the Seurat R package. The columns represent the results from the Louvain algorithm, while the rows represent those generated by the Leiden algorithm. Cell colors indicate the number of common samples between each Leiden and Louvain cluster. When comparing the two algorithms, most clusters were identical in sample composition, including all those containing CTCs: #36, #53, #56, #59, #60 (epi A), #33, #40 (epiB), and #42 (mesenchymal) in the Leiden resolution. However, the Leiden algorithm performed better in separating the earliest embryonic stages by correctly splitting epiblasts and ICM (EpiICM) from the pre-lineages cell types. These were grouped into a single mixed cluster (#33) using Louvain but were resolved into two distinct clusters (#46 and #47) using Leiden. Based on these findings we used the Leiden algorithm throughout the downstream analysis.

Abbreviations: CTC: circulating tumor cell; CTCepiA: epithelial A CTC; CTCepiB: epithelial B CTC; CTCmes: mesenchymal CTC; N: normal breast; LN: metastatic breast cancer lymph nodes; CA: the respective breast cancers related to LN; EVTw8: extravillous trophoblast at 8 weeks; EVTw24: extravillous trophoblast at 24 weeks; STB: syncytiotrophoblast; CTB: cytotrophoblast; Epi/ICM: epiblast/inner cell mass; Prelin: prelineage; PrE: primitive endoderm; TE: trophectoderm; TN, triple negative breast cancer; ER, estrogen receptor-positive breast cancer; HER2, human epidermal growth factor receptor 2-positive breast cancer.


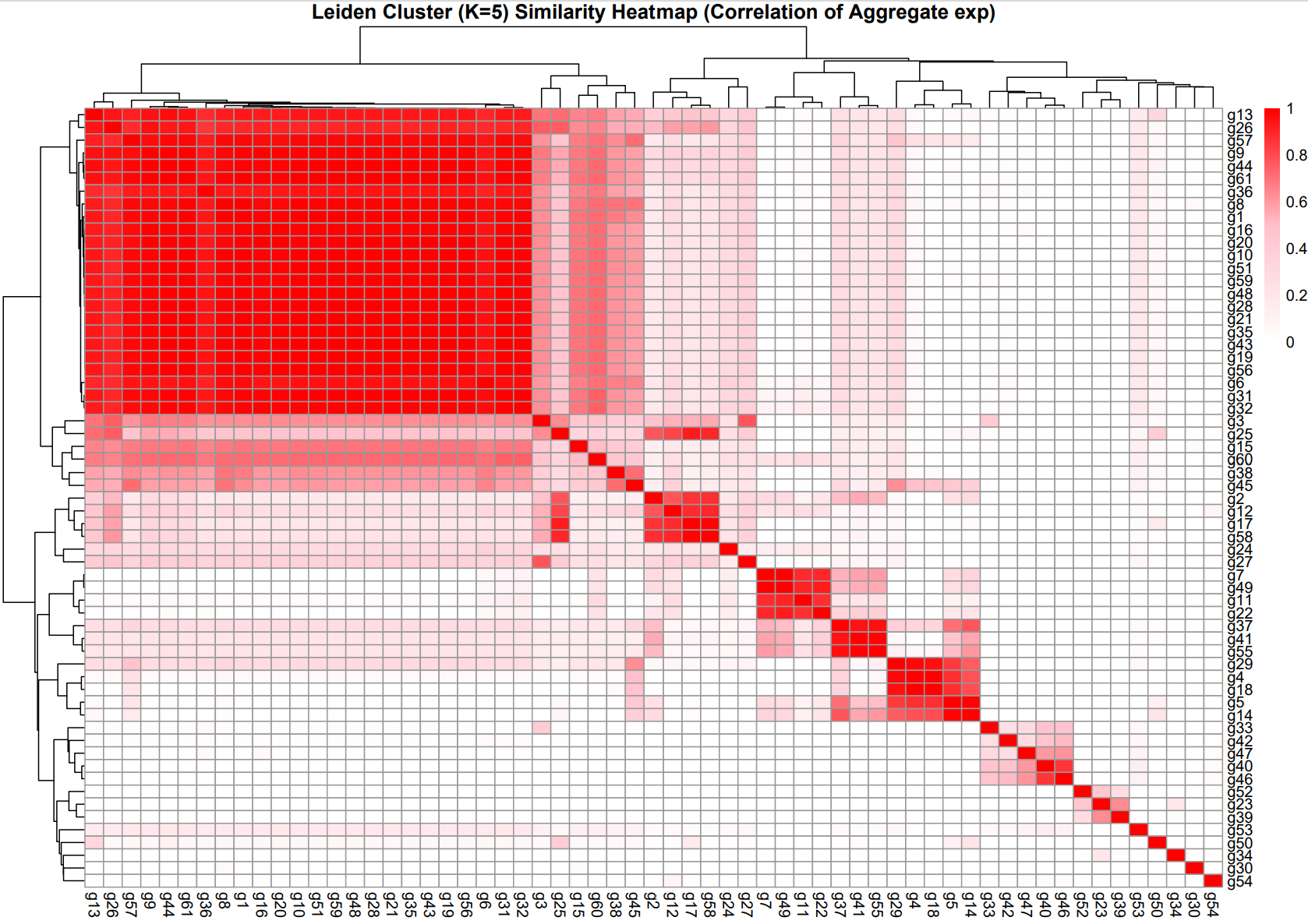


**Supplementary Figure S5. Unsupervised clustering of the integrated CTC/cancer/embryo dataset.**

Using clustering analysis (K = 5 and the Leiden algorithm), we identified finer subgroups among CTCs and their potential correlation with trophoblasts or other embryonic cell types. The epithelial B CTCs are divided into two clusters (#g33 and #g40), with the latter cluster showing stronger association with Epi/ICM and pre-lineage early embryonic cell types. Abbreviations: CTC: circulating tumor cell; CTCepiA: epithelial A CTC; CTCepiB: epithelial B CTC; CTCmes: mesenchymal CTC; N: normal breast; LN: metastatic breast cancer lymph nodes; CA: the respective breast cancers related to LN; EVTw8: extravillous trophoblast at 8 weeks; EVTw24: extravillous trophoblast at 24 weeks; STB: syncytiotrophoblast; CTB: cytotrophoblast; Epi/ICM: epiblast/inner cell mass; Prelin: prelineage; PrE: primitive endoderm; TE: trophectoderm; TN, triple negative breast cancer; ER, estrogen receptor-positive breast cancer; HER2, human epidermal growth factor receptor 2-positive breast cancer.


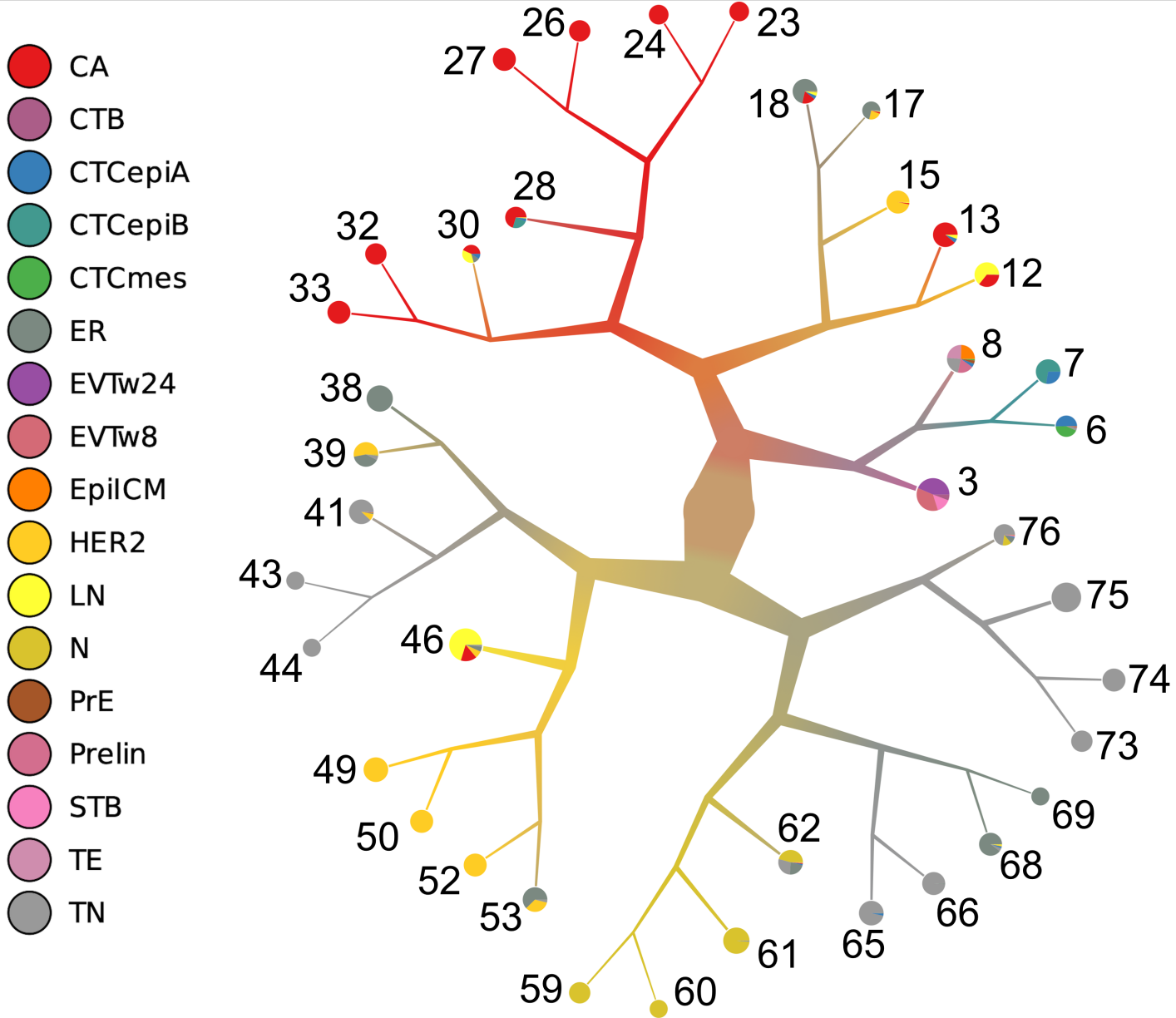


**Supplementary Figure S6.** **Spectral clustering of the integrated dataset (CTC, early embryo, trophoblast, normal, and cancerous breast tissues).**

Clusters are matched by number to their compositions in **Supplementary Table S5**. Nodes were pruned using smart prune set to 0.5 MAD. The color of the clusters and branches correspond to the relative composition of different cell types, while the size of each cluster reflects its numerosity.

Abbreviations: CTC: circulating tumor cell; CTCepiA: epithelial A CTC; CTCepiB: epithelial B CTC; CTCmes: mesenchymal CTC; N: normal breast; LN: metastatic breast cancer lymph nodes; CA: the respective breast cancers related to LN; EVTw8: extravillous trophoblast at 8 weeks; EVTw24: extravillous trophoblast at 24 weeks; STB: syncytiotrophoblast; CTB: cytotrophoblast; Epi/ICM: epiblast/inner cell mass; Prelin: prelineage; PrE: primitive endoderm; TE: trophectoderm; TN, triple negative breast cancer; ER, estrogen receptor-positive breast cancer; HER2, human epidermal growth factor receptor 2-positive breast cancer.


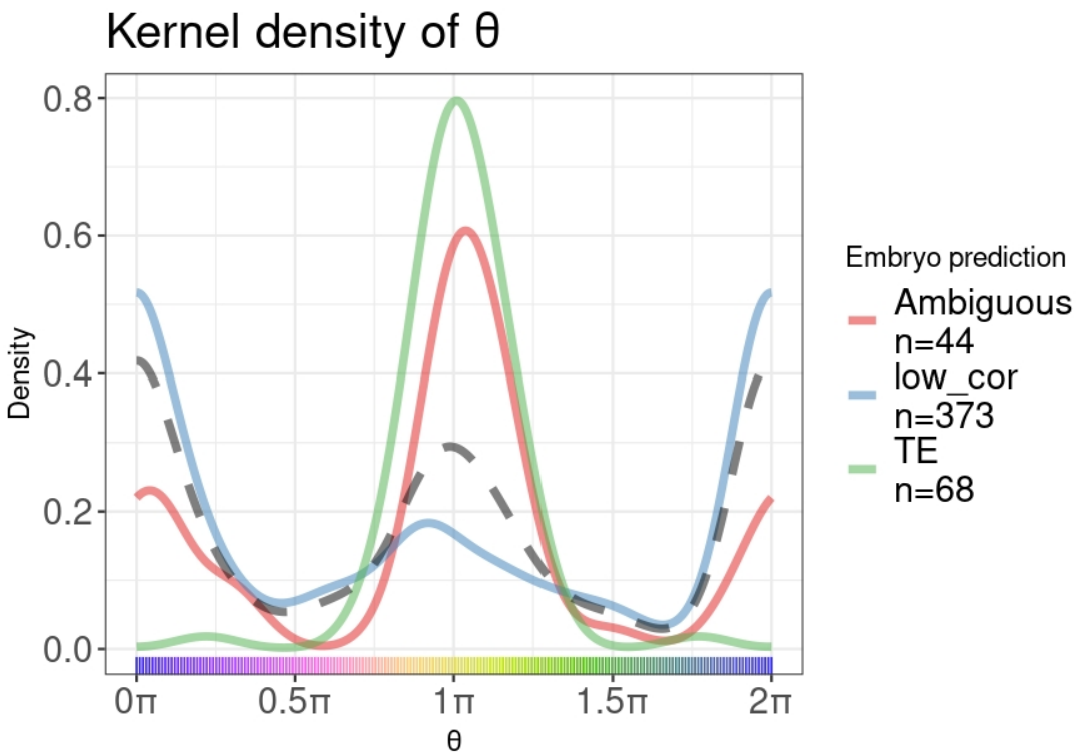


**Supplementary Figure S7. The kernel distribution of cell cycle activity in CTCs based on their similarity to the trophectoderm (TE).**

The TE-like CTCs (green line) were actively engaged in the cell cycle, as indicated by a peak for theta around 1 pi, corresponding to the S-phase). Interestingly, ambiguous calls (i.e., other CTCs assigned to multiple embryonic cell types, red line) also showed cell cycle activation, unlike CTCs with low correlation (no homology to embryonic tissues). Notably, cell cycle genes were excluded from the RNA profiles before mapping to the embryo reference map.


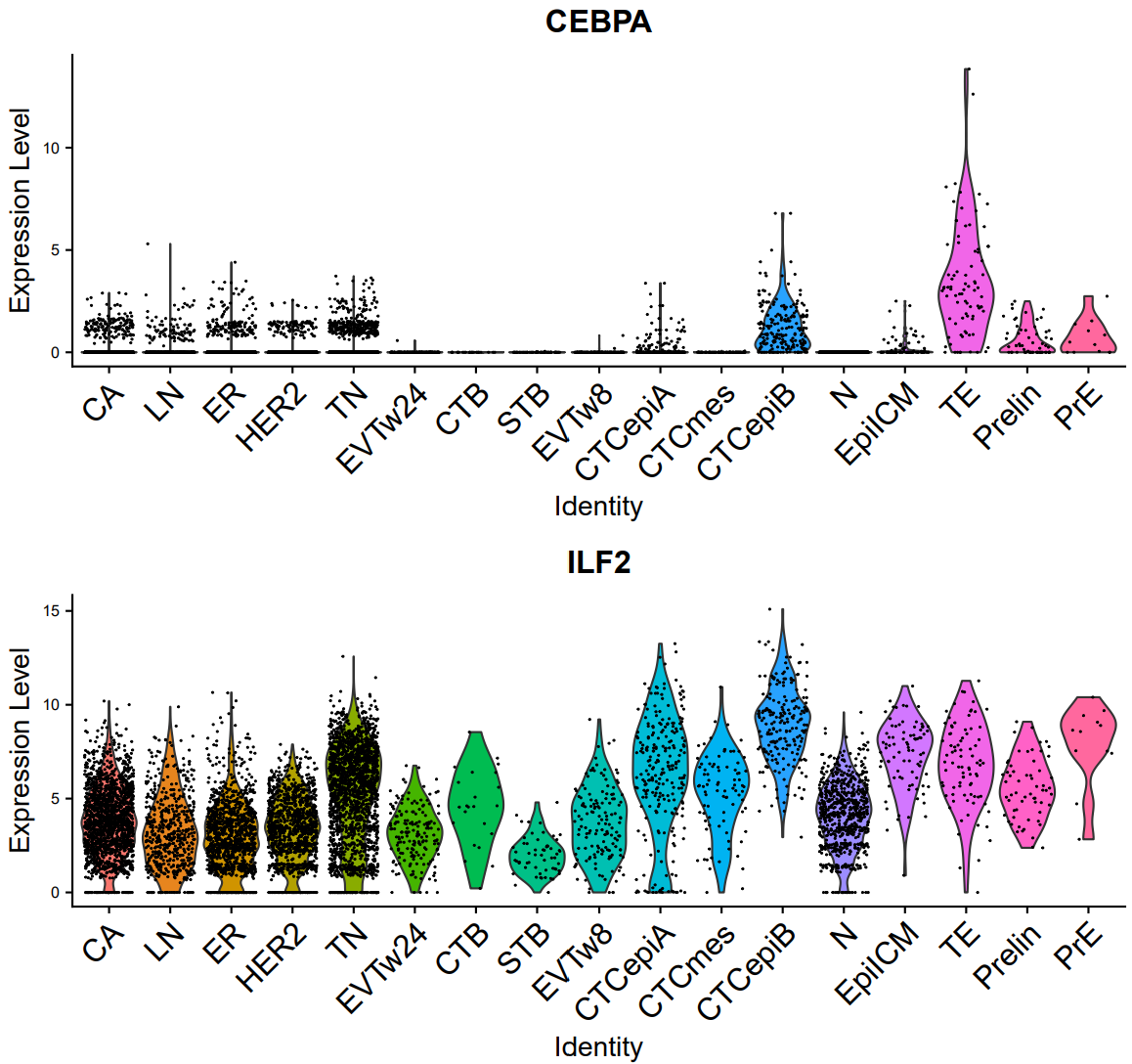


**Supplementary Figure S8. CEBPA and ILF2 are transcription factors highly expressed in CTCepiB and TE.**

The violin plots show the expression levels of the CEBPA and ILF2, which are upregulated in epiB CTC and TE. CEBPA expression is more specifically restricted to CTCepiB and TE. Abbreviations: CTC: circulating tumor cell; CTCepiA: epithelial A CTC; CTCepiB: epithelial B CTC; CTCmes: mesenchymal CTC; N: normal breast; LN: metastatic breast cancer lymph nodes; CA: the respective breast cancers related to LN; EVTw8: extravillous trophoblast at 8 weeks; EVTw24: extravillous trophoblast at 24 weeks; STB: syncytiotrophoblast; CTB: cytotrophoblast; Epi/ICM: epiblast/inner cell mass; Prelin: prelineage; PrE: primitive endoderm; TE: trophectoderm; TN, triple negative breast cancer; ER, estrogen receptor-positive breast cancer; HER2, human epidermal growth factor receptor 2-positive breast cancer.


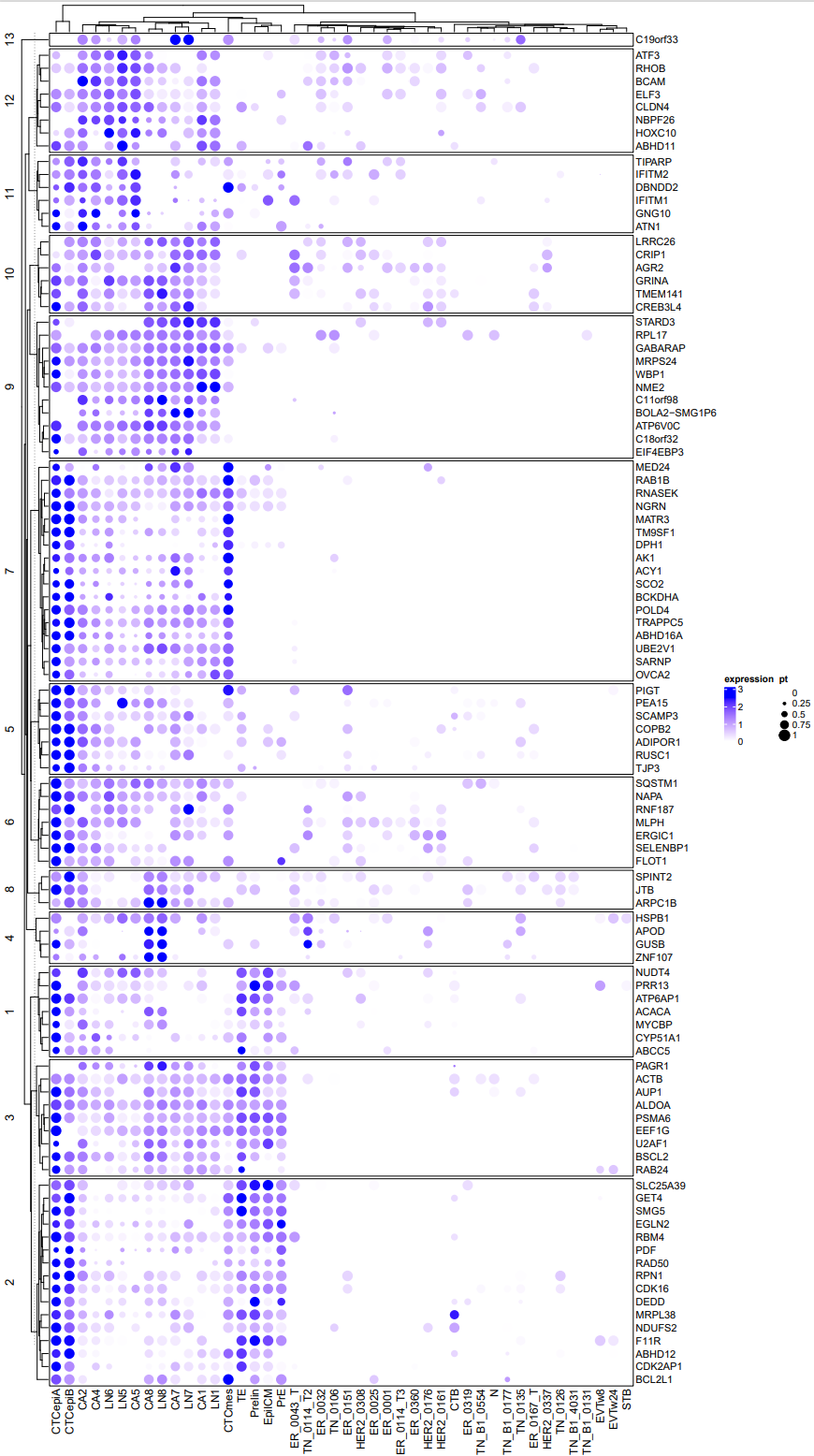


**Supplementary Figure S9.** **Identification of RNA modules relevant to metastatic evolution. Differentially expressed genes were identified by comparing CTCs, metastatic lymph nodes (LN), and their respective primary tumors (CA) with non-metastatic cancer and normal breast epithelial cells*.***

The suffixes after CA, LN, ER, TN, HER2 indicate different cancer samples within the cancer subgroups.

Several gene modules displayed expression patterns associated with the metastatic process (listed in **Supplementary Table S7**). In Box 3, two genes (ALDOA and PSMA6) were upregulated in CTCs, early embryo cells with trophectoderm (but not the trophoblasts), cancer cells derived from metastatic lymph nodes, and their corresponding primary tumors.

Abbreviations: CTC: circulating tumor cell; CTCepiA: epithelial A CTC; CTCepiB: epithelial B CTC; CTCmes: mesenchymal CTC; N: normal breast; LN: metastatic breast cancer lymph nodes; CA: the respective breast cancers related to LN; EVTw8: extravillous trophoblast at 8 weeks; EVTw24: extravillous trophoblast at 24 weeks; STB: syncytiotrophoblast; CTB: cytotrophoblast; Epi/ICM: epiblast/inner cell mass; Prelin: prelineage; PrE: primitive endoderm; TE: trophectoderm; TN, triple negative breast cancer; ER, estrogen receptor-positive breast cancer; HER2, human epidermal growth factor receptor 2-positive breast cancer.


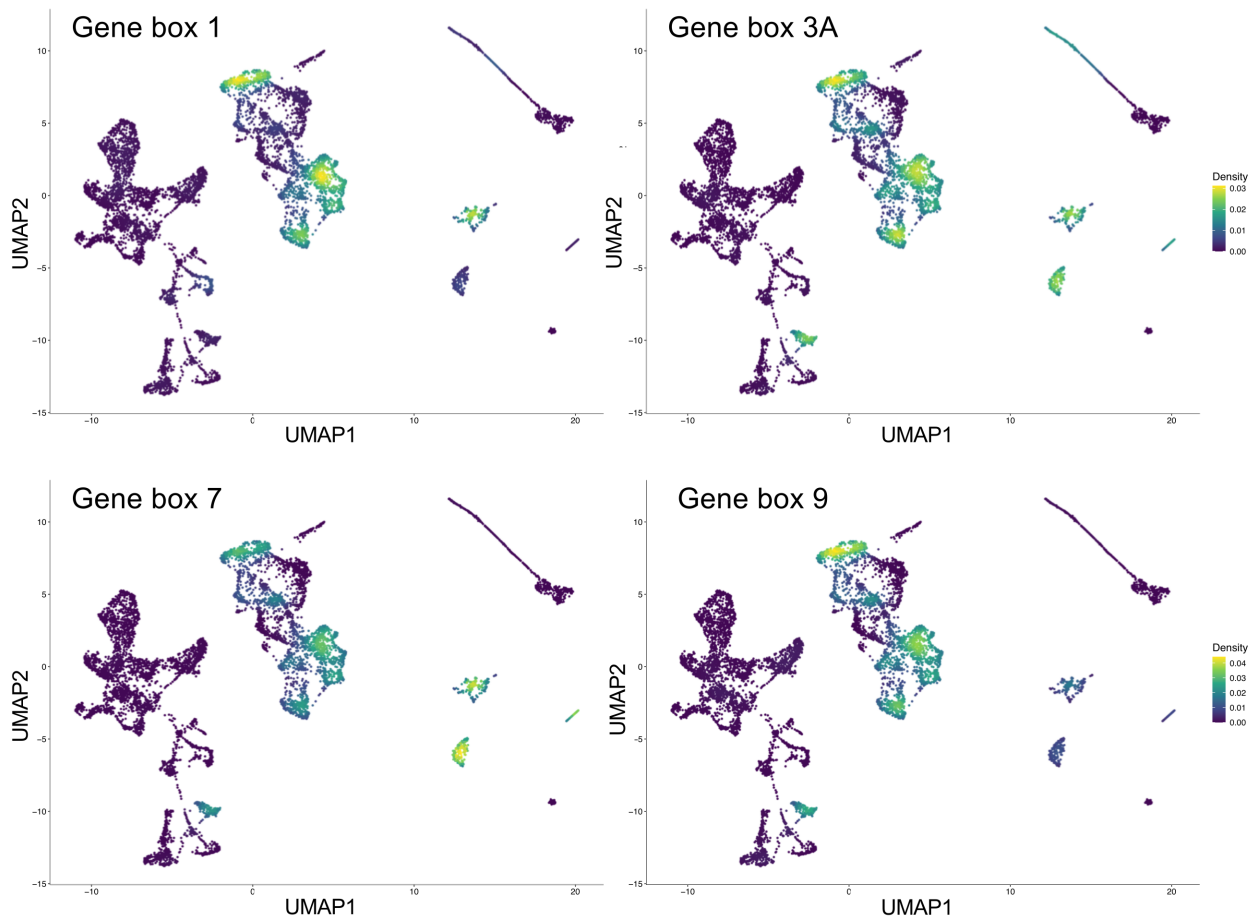


**Supplementary Figure S10.** **UMAP visualization of CTC and metastatic RNA modules.**

RNA modules specifically upregulated in CTCs, breast cancer metastatic lymph nodes (LN), and primary tumors with metastatic lymph nodes (CA)**.**

Genes for RNA modules in boxes 1, 3A, 7, and 9 (**Supplementary Table S7**).


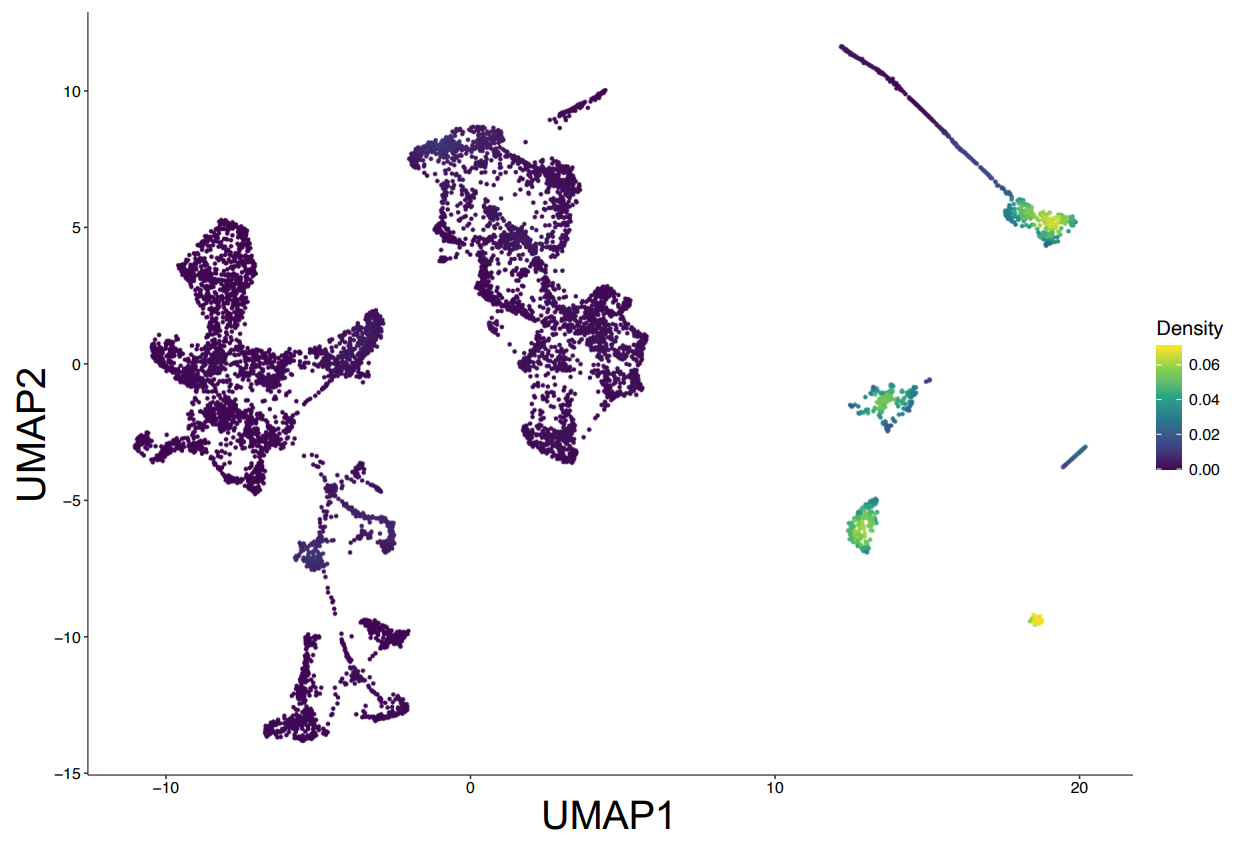


**Supplementary Figure S11.** **UMAP visualization of CTC and trophoblast RNA modules.**

RNA modules specifically upregulated in CTCs and trophoblasts (*RBM34*, *ATXN7*, *GNS*, *RELL1*) (**Supplementary Table S7**).


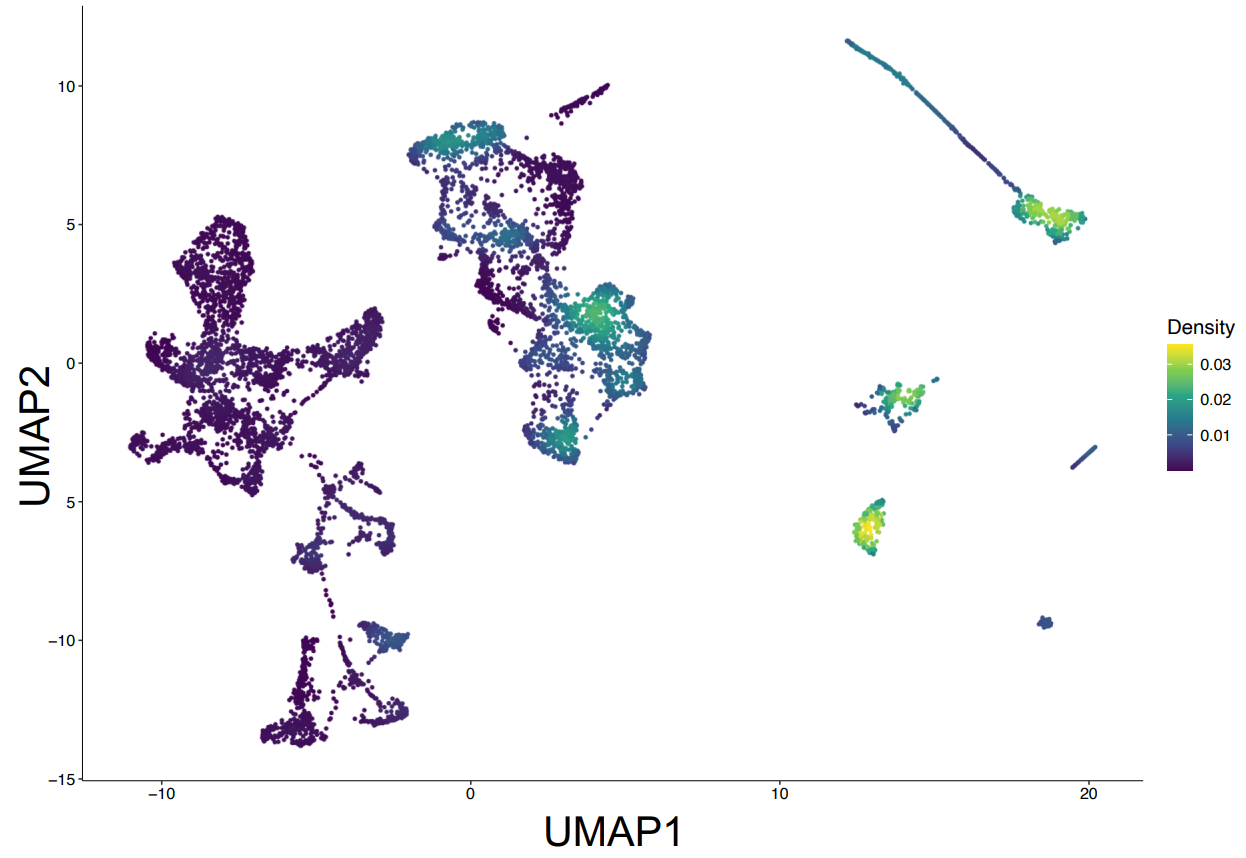


**Supplementary Figure S12. UMAP visualization of CTC, metastatic lymph nodes, and trophoblast RNA modules.**

Genes specifically upregulated in CTCs, metastatic lymph nodes, primary tumors with metastatic lymph nodes, trophoblasts, and early embryos (*GET4*, *RAB24*, *VAT1*, *F11R*, *BAIAP2L1*, *GDF15*)**.**

**Supplementary Table S1.** Single-cell RNA-seq (scRNA-seq) profiles of bona-fide keratin-positive and aneuploid CTCs.

[Excel]

**Supplementary Table S2.** Distinctive genes for epithelial A CTCs.

[Excel]

**Supplementary Table S3.** Distinctive genes for epithelial B CTCs.

[Excel]

**Supplementary Table S4.** Distinctive genes for mesenchymal CTCs.

[Excel]

**Supplementary Table S5.** Spectral Clustering.

[Excel]

**Supplementary Table S6.** The Normal breast/cancer/CTC lineages obtained using minimum spanning tree and pseudo-time.

[Excel]

**Supplementary Table S7.** CTC, trophoblast, and metastatic RNA modules.

[Excel]

**Supplementary Table S8.** Cell cycle genes used as markers of the G2/M and S phases.

[Excel]

**Supplementary Table S9.** Cluster resolution using Louvain algorithm (K = 5).

[Excel]

**Supplementary Table S10.** Cluster resolution using Leiden algorithm (K = 5).

[Excel]

**Supplementary Table S11.** Louvain Leiden Common samples.

[Excel]
